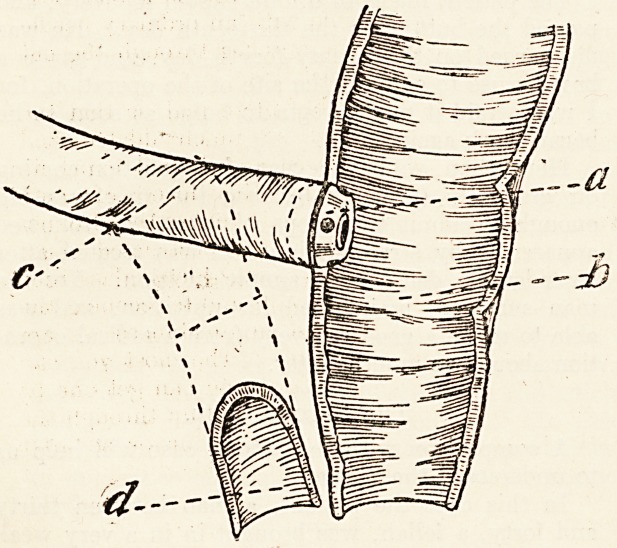# Strangulated Hernia

**Published:** 1911-03-04

**Authors:** Owen Richards

**Affiliations:** Professor of Clinical Surgery, Egyptian Government School of Medicine, Cairo.


### Hospital Clinics.

### STRANGULATED HERNIA.

By OWEN RICHARDS, M.Ch.Oxon., F.R.C.S., Professor of Clinical Surgery, Egyptian Government School of Medicine, Cairo.

Gentlemen,—There is no principle in surgery more emphatically taught than the necessity of operating at once on all cases of strangulated hernia. In these cases, more than any others that you are likely to meet with in practice, the life of the patient depends absolutely on your prompt interference. And although I am bringing before you to-day two cases which may be considered exceptions—for they both survived for some weeks without anything which can be called an operation—yet on consideration you will see that they are really the kind of exceptions which prove the rule.

I will first describe the cases to you, and we will then discuss the practical lessons which can be drawn from them. I cannot show you the patients, for one is dead, and the other has been discharged cured.

### The First Case.

The first case was that of a fellah about thirty years old, who walked into my out-patient room with a large mass bulging from his left groin. When he stripped, this was revealed as an earthen pot tied on with string and half full of fæces. Underneath it was a soft mass about the size of a fist, reducible into the abdomen through a round opening in the wall just above the middle of Poupart's ligament. On its summit was a round hole, admitting the finger, lined with mucous membrane, and discharging fæces.

The history, which I need hardly warn you is quite unreliable as to dates, was that three years ago he noticed a small rounded swelling in this situation, reducible into the abdomen. Three months ago this reached the size of an orange, and he could no longer reduce it. Soon afterwards he developed constipation, vomiting, and local pain. This lasted on and off for a fortnight; finally it became worse, and a doctor made an incision into the most prominent part of the swelling and let out a quantity of fæces.

The symptoms then subsided, and since then he had passed fæces both by the anus and the wound. Owing to the projection of the mass he found that he could keep himself clean by tying on the pot; and he has been in this condition some two months. He was admitted, and three days later I made an incision encircling the opening, turned up the edges of the inner circle of skin, and sewed them tightly together. This was wrapped in gauze and the surroundings cleaned. The mass was then opened up, and found to consist of a sac containing two loops of intestine descending through a hernial opening. One was only adherent at its apex; it was freed and returned. The other, on the convexity of which was situated the fistula, was adherent to itself, to a tag of omentum, and to the sac-wall; it was impossible to clear it. On tracing it upwards one end was fairly free, and could be mobilised; the other was so firmly bound down inside the abdomen that without enlarging the wound greatly it was impossible to free it. Accordingly the coil in the sac, seven inches long, and the omentum were excised. The movable end was brought down to the fixed one and joined to it just inside the abdomen by a Murphy's button, reinforced by a Lambert's suture. The abdominal muscles were then freed and drawn down to Poupart's ligament.

### The Moral of the Case.

The patient made an uninterrupted recovery, and passed the button on the thirteenth day. He was discharged on the twenty-fourth day, and given a broad truss to support the site of the operation, for I was afraid it might begin to bulge as soon as he began work again.

Here, then, we have a case of obstruction coming on gradually (possibly because the ring was big enough to admit two loops of intestine), followed apparently by strangulation. It was treated after considerable delay by a simple incision. Yet the man survived, maintained his nutrition, and was able to make a good recovery from a radical operation about two months later.

### The Second Case.

A comparison with the second case will help us to understand the reason.

In this case the patient, a man between thirty and forty, a fellah, was brought in in a very weak condition. In the upper part of the right side of the scrotum were three irregular fistulæ, the biggest 1½ feet long, through which all his fæces passed. He had a purpuric rash on the abdomen, was much emaciated, and had a poor pulse. He stated that he had occasional vomiting and colic.

His history—also unreliable—was that three weeks ago he developed pain and swelling all over the right side of the abdomen up to the liver. Four days later the skin of the scrotum sloughed, and there was a discharge from it of pus and fæces. The symptoms then subsided, and since that time all his fæces have passed from the scrotal opening, none from the anus. There was always a certain amount of colic and rumbling in the right side. He was admitted, and ordered nourishing diet and nutrient enemata, with the idea of getting him into a fit state for operation. However, on the third morning he developed acute obstruction, with a distended abdomen, visible peristalsis of large coils of intestine, colic, and vomiting. Under somniform the scrotum was slit up, revealing two separate open ends of intestine. An attempt was made to pass tubes up these, but no fæces could be got to flow. Accordingly he was given chloroform, the incision carried upwards, and the abdomen opened. Two pieces of intestine were found descending into the scrotum, of which one was enormously hypertrophied and distended, the other atrophied so as to be almost transparent, and having a very small lumen. The upper distended intestine was first emptied by passing a wide tube into its lumen from below. The lower shrunken intestine was divided a few inches above the ring, its upper end was implanted with a Murphy's button into the side of the upper intestine, its lower end was closed inside the abdomen, so as to form a pouch opening downwards into the wound. The scrotal lower end of the upper intestine was left open as a safety valve. So that there was a T-shaped junction with all arms open, and fæces descending could either pass through the button into the lower intestine or directly to the surface in the scrotal wound.

After operation vigorous borborygni were heard, and a little fæcal matter passed per anum. Next day the patient was weak, but took his feeds, did not vomit, and his bowels were opened seven times naturally. No fæces came from the wound.

On the third day he sank and died, apparently of acute peritonitis. No satisfactory post-mortem was made, but my own opinion is that the atrophied gut probably yielded at the junction.

As to the use of Murphy's button in these cases, I never use it if it is possible to use simple suture. But in the first case it was mechanically difficult to get at the fixed end of intestine to suture it, in the second the atrophied intestine would not have held sutures. With the button it was risky enough, too much so, as events showed; but I trusted to the opening in the scrotum to prevent any strain falling on the junction. Moreover, I felt that unless some fæces were diverted into the lower intestine, the patient's condition would go on getting worse, even if the obstruction were relieved.

### Some Deductions.

The cause for this striking difference in the condition of these two patients, and the results obtained, are, I think, threefold.

The first cause was that one had a "fæcal fistula," and the other an "artificial anus." In the one that died there was an artificial anus, or rather an accidental one, for it was not due to art. That is to say, all the fæces passed from the wound, none went on into the lower intestine. In the course of three weeks, if we are to accept the patients' dates, atrophy of the disused intestine had become very marked; it was unfit for suture, and probably gave way with a Murphy's button. Such fæces as passed along it to the anus must have been propelled by pressure from behind rather than by any effective peristalsis. I find a difficulty in believing that all this can have taken place in three weeks, but at any rate it did take place, and was probably the principal cause of the failure of the operation.

In the first case, on the other hand, there was a fæcal fistula only. Some fæces continued to pass into the lower intestine; there was consequently no atrophy, and it was possible to make a sound junction.

The second cause, depending on this, was the difference in nutrition. It has been stated that an artificial anus anywhere more than two feet above the ileocæcal valve will lead to death from starvation. Most hernial loops are higher up than this; where these actually were I did not determine. But the result was that the man with the artificial anus was visibly on the edge of the grave, the man with the fæcal fistula was walking about, and stood up in front of me at out-patients' and told his story quite cheerfully.

The third cause was the nature of the opening. In the case that lived, the opening was direct, mucous membrane protruded from it, and there was no obstruction. In the case that died the bowel communicated with the surface by tortuous, narrow fistulæ, and there was obstruction with its consequent poisoning.

### A Note on Operative Interference.

Now all these three causes depend essentially on one—the manner in which the opening was made.

In the first case a simple short incision had been made in the bowel, as it lay against the sac wall, distended and strangulated, before its vitality had been entirely lost. This let out the contents, diminished pressure and congestion, relieved the strangulation, allowed fæces to pass, and left the patient with a fistula in the side of a loop of bowel, which preserved its living continuity as a loop.

In the second case nature unassisted made an anus. That is to say, the greater part of the loop and the skin over it sloughed away. This interrupted the continuity of the loop, and consequently caused starvation of the patient and atrophy of the bowel below. At a later date the cicatricial contraction round the opening caused a progressive obstruction.

So that we are led to the conclusion that of these two cases of unoperated strangulation, one survived because it was incised, the other died because it was not.

Now most of you will shortly be in practice in the provinces, and you will pretty often meet with urgent cases which have to be dealt with single-handed and at once. I have told you of two cases of strangulated hernia which survived neglect; how many do you suppose have been neglected in the same time and have not survived? Probably they bear the same proportion to these that the shells on the shore do to those in the sea.

And you must consider that the victims of this condition are not commonly old men, or sick men, who can easily (from the economical standpoint at least) be spared; they are like these patients—men in the prime of life, fathers of families, capable of living long and useful lives if you can save them, certain to die if you cannot. You all know how to operate on them. The question is, Can you always operate? and the answer is, Always. By using spinal anæsthesia as you see it used here, you can be independent of an anæsthetist, and the outfit will go in your pocket. You need no instruments or ligatures which will not go in your pocket-case. And if you cannot get iodine, or lysol, or spirit, you can always use boiled water and soap, and trust to luck.

### Conclusions.

But supposing you have none of these things. Of course, you can try taxis if the case is a recent one; and you are more likely to succeed if you put the patient with his head lower than his legs. But if it fails, as it commonly does, you need not apply yourselves to making pious remarks of a fatalistic character—there are always plenty of people to do that. You can always get a dirty razor, and with that and a little courage you can make a fæcal fistula. And if you do that, some few, at any rate, of these patients will survive; I have just told you of one.

This is essentially the proceeding described in the most sensible book I know of, Jacobson and Rowland's *Operations of Surgery* (vol. ii. p. 49). It is recommended there for cases where the loop is hopelessly gangrenous and the patient moribund. Of a series of such cases treated in this way one in ten survived. But you are dealing, not with cases where the loop is known to be gangrenous, but with cases where it may be capable of recovery, and the intestine above capable of emptying itself.

So that the mortality need not be so high—in any case it can hardly be above 100 per cent., which is the death-rate of cases left alone. Even the second case I quoted to you died in the end, and would have died without the operation.

So if you can save even one case in ten, you may be content to submit to the charge of having killed the other nine.

Of course, simple incision of a strangulated hernia is hardly surgery at all. Perhaps that is why books on the subject do not refer to it. But there is very little fear of its failing to relieve the obstruction. When you have let out the contents of the loop and incised the œdematous bowel, you are pretty sure to get sufficient reduction of the swelling to allow liquid fæces to pass—and the fæces always are liquid. And it is the obstruction rather than anything which happens to the bowel which kills the patient.

But if adhesion has not occurred at the neck, it is conceivable that the loop, thus relieved, might slip back into the abdomen and discharge its contents there. I do not know if this is a real risk, but it ought to be possible with an ordinary needle and thread to take a few stitches through the cut edges, and so guard against this.

Another danger, probably more real, is that the intestine above has been so much dilated and poisoned that it is paralysed and cannot expel its contents. As you know in other forms of acute obstruction it is no use simply to relieve the obstruction, one must also empty the obstructed coils. This is often done by introducing a long glass tube through an incision in the wall, and telescoping the full coils on to it. In the circumstances we are considering that is impossible. The most you can do is to try and pass a tube if you can get one (it need not be clean) into the gut and up through the ring if no fæces appear for an hour or so after incision. I tried and failed to do this in the second case. But I doubt its being much use if the gut is really in a state of paralytic distension; the patient will probably die whatever you do.

So do not think that this method is a good one if you can possibly do anything better. The practical lesson I want you to learn from these cases is that if you have no means of doing even the simplest operation, and no chance of moving your patient in a reasonable time, you may still sometimes save a life by simply making a cut with a razor.

## Figures and Tables

**Figure f1:**